# Supplementary material for: The barley leaf rust resistance gene Rph3 encodes a predicted membrane protein and is induced upon infection by avirulent pathotypes of Puccinia hordei
Source: Nat Commun. 2022 May 2;13:2386. doi: 10.1038/s41467-022-29840-1 (PMC9061838; doi:10.1038/s41467-022-29840-1)
Supplement: Supplementary file 1 — Supplementary Information [file 41467_2022_29840_MOESM1_ESM.pdf]

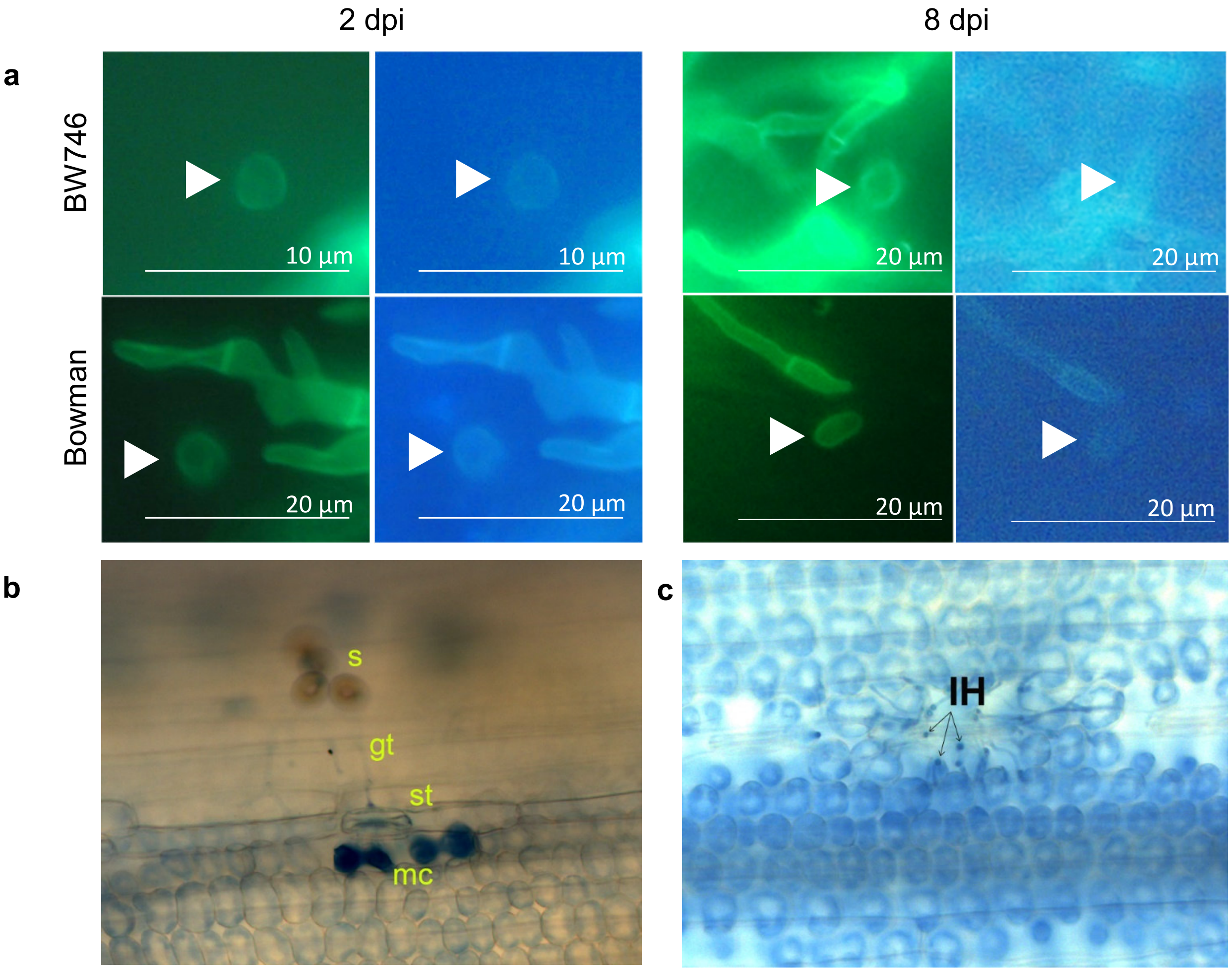

Supplementary Figure 1. Infection of resistant and susceptible barley lines with *Puccinia hordei* pathotype 5453 P+. **a** At both two dpi and eight dpi, haustoria were present in infected cells of both BW746 and Bowman (green images on left; arrowheads show WGA-FITC stained haustoria). Haustorium-containing plant cells at these same infection sites were not associated with autofluorescent cell death when observed under UV light, in either line (blue images on right). **b** Germinated spores (s) can be seen on the leaf surface of BW746 that have formed germ tubes (gt). One of these infection sites has located a plant stomate (st) and four underlying mesophyll cells (mc) have strongly stained with trypan blue, indicating membrane changes in these mesophyll cells in response to fungal infection. **c** In contrast to BW746, all the mesophyll cells at a fungal infection site on Bowman show no stain uptake indicating little changes in membrane structure of infected cells. Thread-like fungal infection (IH) can be seen at this infection site ramifying between host mesophyll cells. The experiment was repeated three times with similar results.

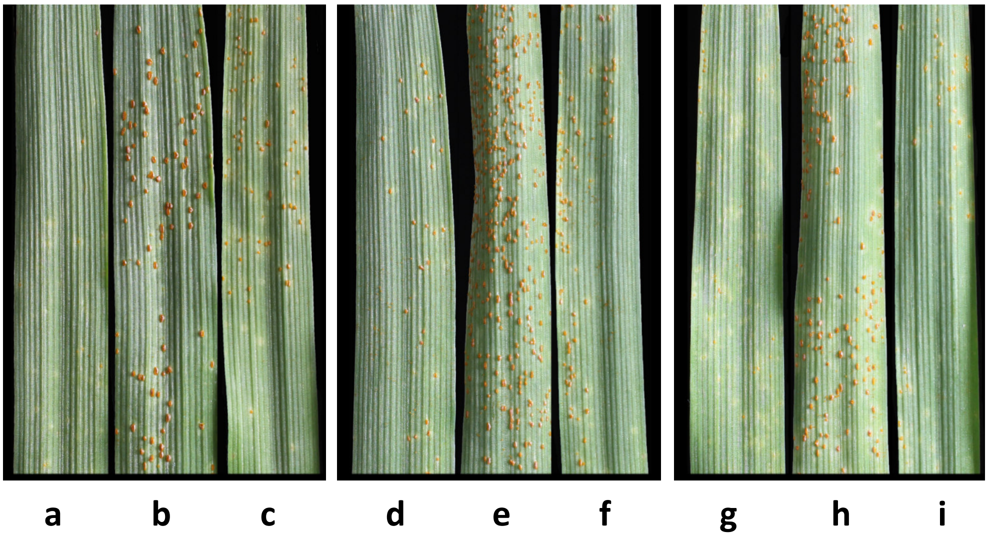

Supplementary Figure 2. *Rph3* is incompletely dominant. Infection types observed in resistant, susceptible parents and offspring. (*A*) Scarlett (*Rph3/Rph3*); (*B*) Tallon (*rph3/rph3*); (*C*) Scarlett x Tallon F_1 (*Rph3/rph3*); (*D*) Alexis (*Rph3/Rph3*); (*E*) Sloop (*rph3/rph3*); (F) Alexis x Sloop F_1 (*Rph3/rph3*); (G) BW746 (*Rph3/Rph3*); (*H*) Bowman (*rph3/rph3*); (*I*) BW746 x Bowman F_1 (*Rph3/rph3*). The inoculation was performed on 10 – 15 plants of each material with similar results.

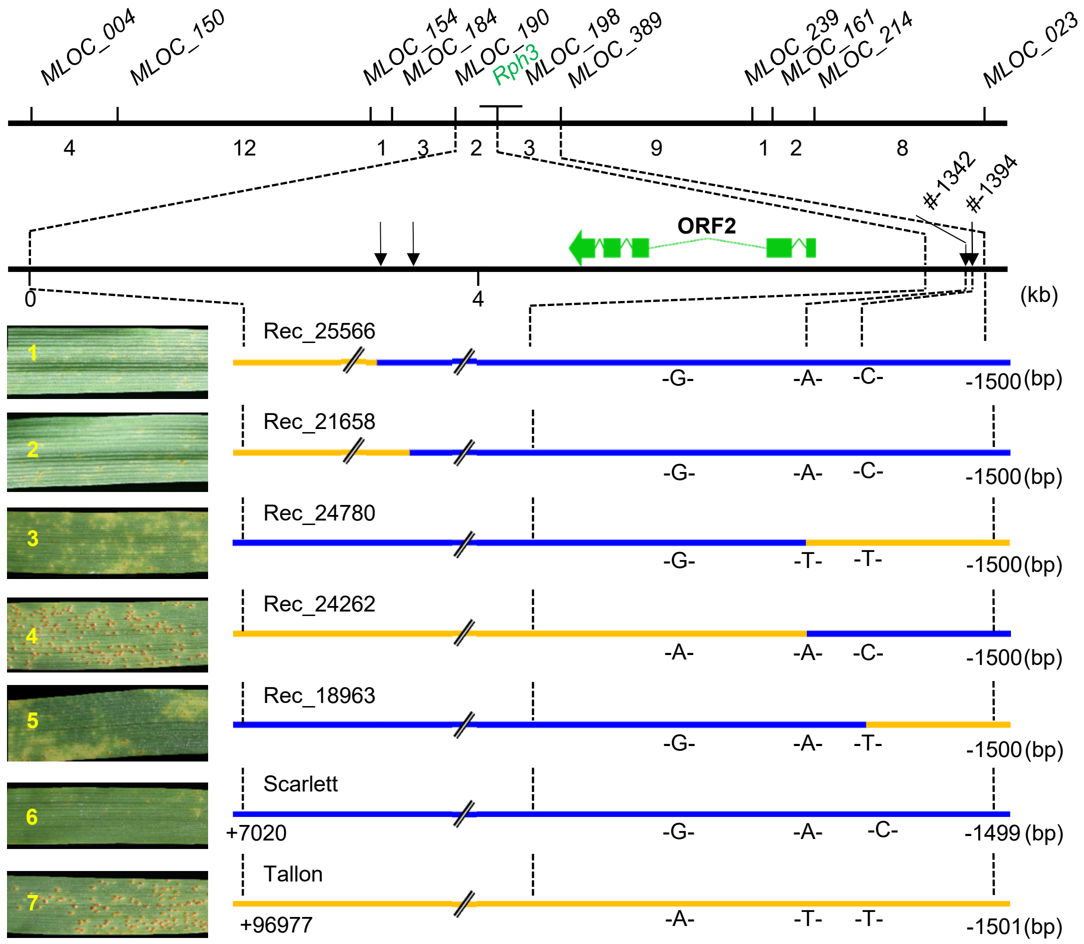

Supplementary Figure 3. Locations of recombination events in the vicinity of the *Rph3* gene were confirmed by Sanger sequencing. The genetic and physical map of the *Rph3* locus delimited by the closest flanking markers MLOC_190 and MLOC_389. The crossover occurred between the nucleotide position -1342 and -970, resulting in genotype changes from *Rph3* (represented by blue part) to *rph3* (represented by the orange part) in the plant numbered 24262 and vice versa in the plant numbered 24780; the crossover between the nucleotide position -1342 and -1394 resulted in the genotype change from *Rph3* to *rph3* in the plant numbered 18963. The infection type observed in resistant (1), susceptible (5) parents, and three families (2, 3, 4) carrying critical recombinants upstream of *Rph3*. The inoculation was performed on 15 plants of each material with similar results.

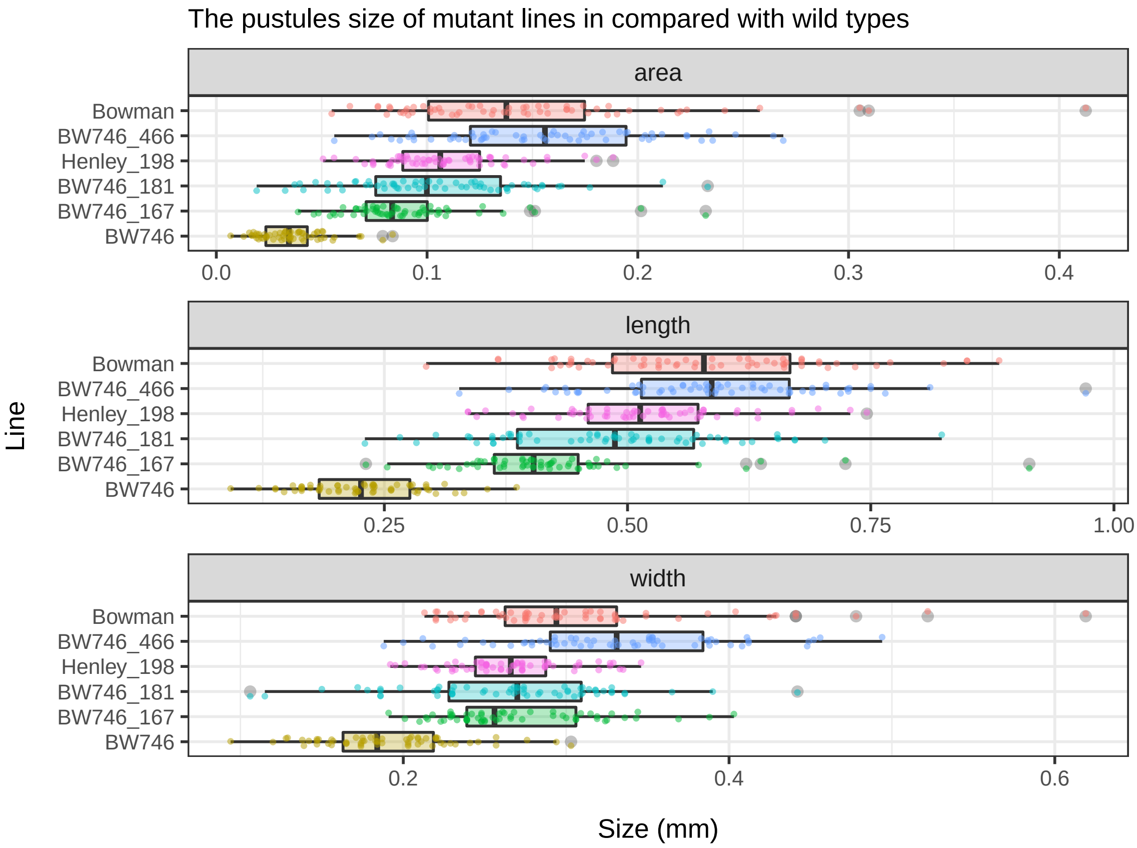

Supplementary Figure 4. Uredinium size of *P. hordei* pathotype 5453 P+ in the knock-out mutants. The data for each mutant were gathered from 60 uredinia (3 leaves × 20 uredinia/leaf) using the ImageJ software. In each box plot, the band within the box indicates the median; the box indicates the first and the third quartile; the whiskers indicate ± 1.5 × interquartile range; the minimum value excluding outliers is shown at the beginning of the left whisker and the maximum value excluding outlier is shown at the end of the right whisker.

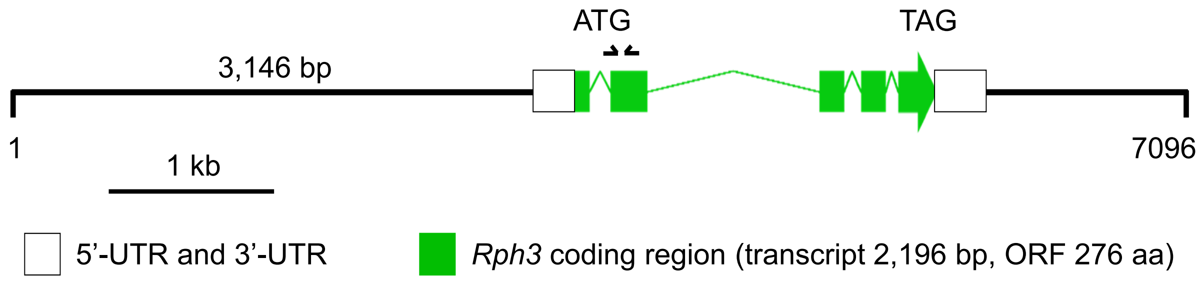

Supplementary Figure 5. The transgene sequence comprised a 7,096 bp genomic fragment harbouring the *Rph3* coding sequence with its native promoter. Exons are shown as green squares with the arrow in the last exon showing the transcript direction. The *Rph3* gene (including introns and exons) was 2,196 bp. The 5’-UTR and 3’-UTR of 254- and 292-bp, respectively, are shown as white squares. Black arrows illustrate the position of the diagnostic MLOC_400 marker for the *Rph3* gene.

a

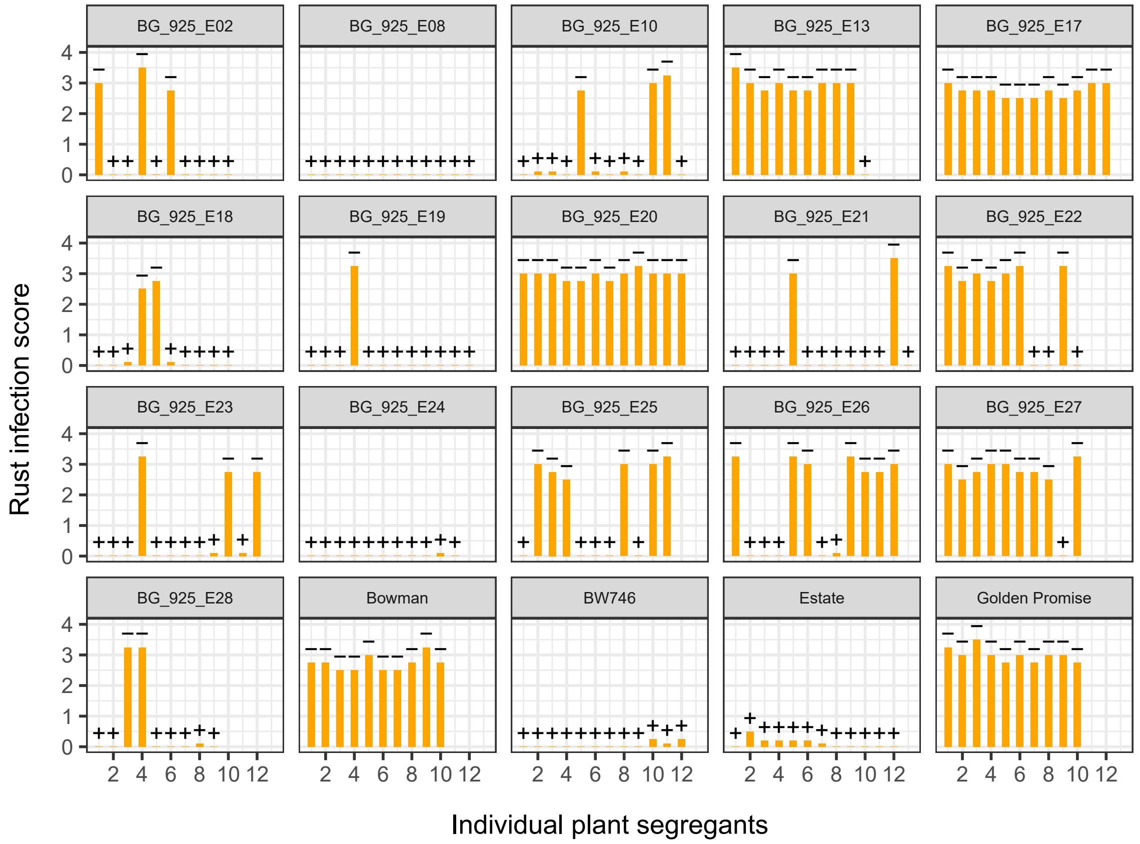

b

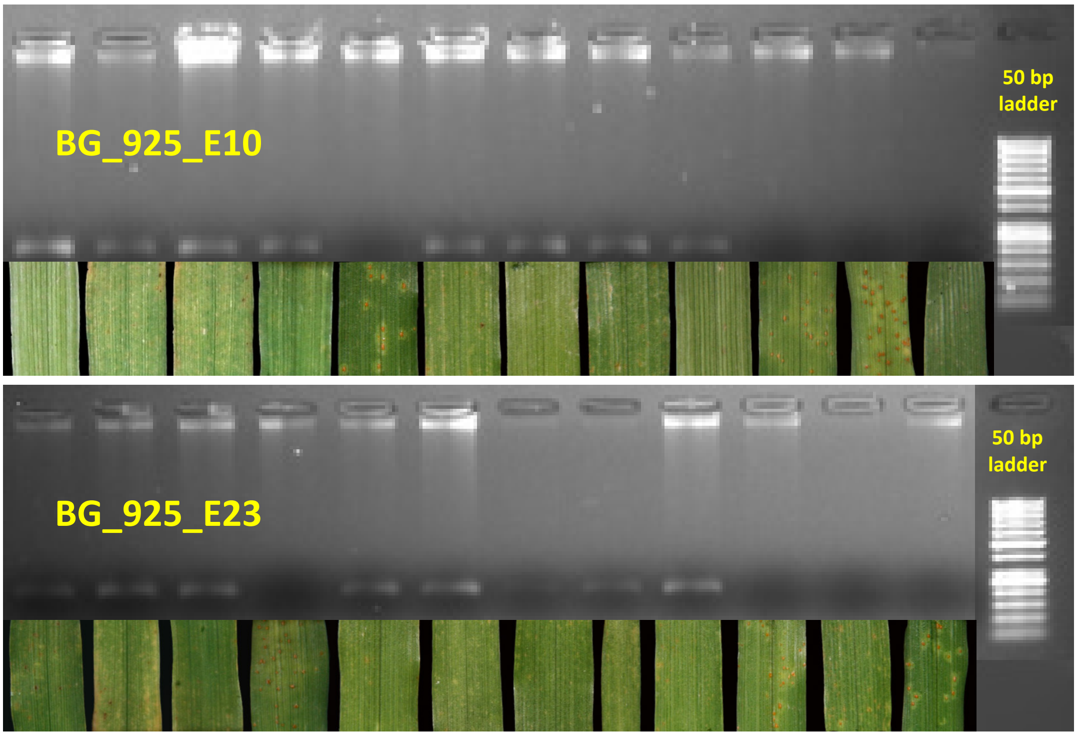

Supplementary Figure 6. Progeny tests of *Rph3* transgenic plants. **a** The responses *to P. hordei* pathotype 16-3 of 16 independent transformants (n=1). The transgene plants with (+) and without (-) *Rph3* were identified by the dominant marker MLOC_400. **b** Phenotypes of some transgenic gene families. The bands on the agarose gel show presence of the *Rph3* marker. The image under the agarose gel shows the leaf rust response. The experiment was conducted without replications.

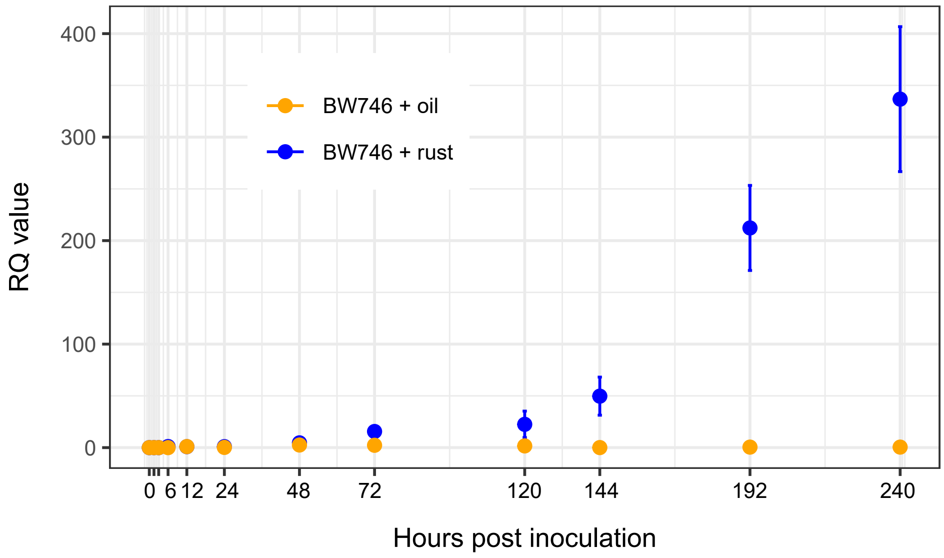

Supplementary Figure 7. Expression profile of the *Rph3* allele in an extended time course. Blue dots indicate the expression of *Rph3* for the treatment challenged with *P. hordei* pathotype 5453 P+ and by orange dots for the mock inoculation. Relative quantification (RQ) value was calculated using the delta-delta method with RQ = $2^{-\Delta\Delta Cq}$. Values represent means ± SD (shown as the error bars) (n=3).

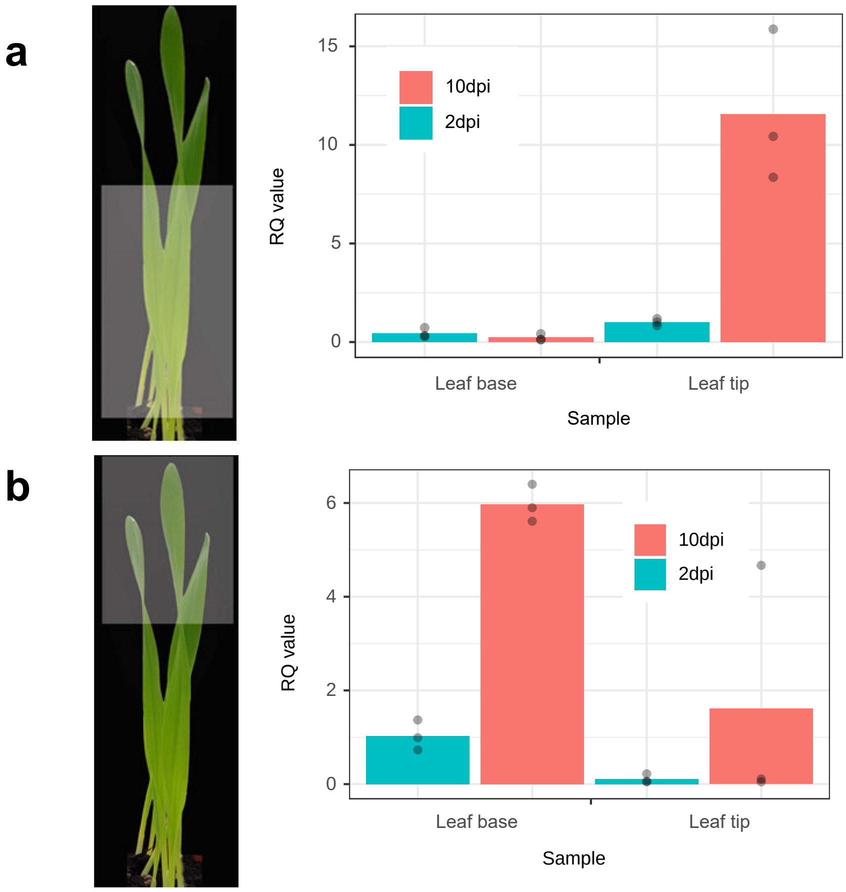

Supplementary Figure 8. Localized expression of the *Rph3* allele in cv. BW746 challenged with *P. hordei* pathotype 5453 P+. Distal (**a**) and proximal (**b**) leaf sections were inoculated separately as illustrated, and expression in the respective host tissues was determined. Relative quantification (RQ) value was calculated using the delta-delta method with RQ = $2^{-\Delta\Delta Cq}$ (n=3).

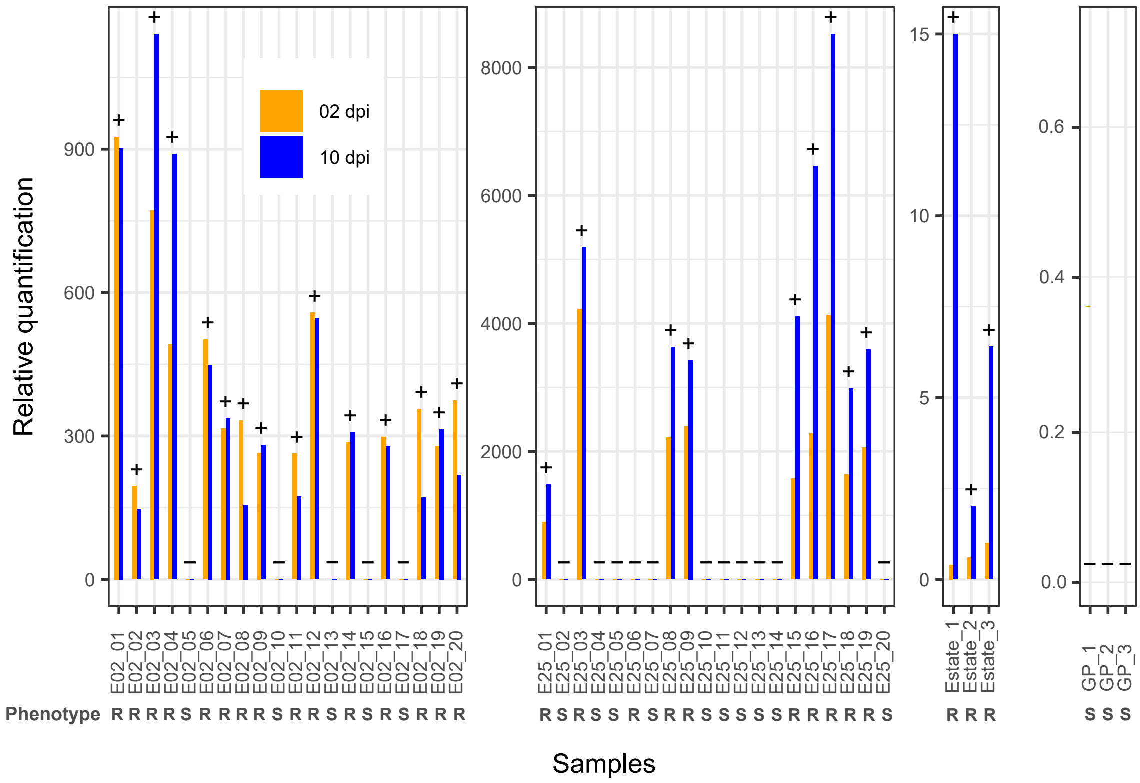

Supplementary Figure 9. Expression of the *Rph3* allele in transgenic and control materials challenged with *P. hordei Rph3*-avirulent pathotype 16-3. Expression levels were determined across the Rph3-segregating T_1 families E2 (**a**) and E25 (**b**), cv. Estate (**c**) as positive control, and cv. Golden Promise (**d**) as negative control. Line E25-06 is the only exception with a negative PCR indicative of not carrying the transgenic gene but showing a resistant phenotype. This is probably due to the line scape from rust pathogen during the inoculation. The +/- symbols represent the presence/absence of the *Rph3* allele based on the diagnosis marker MLOC_400. Relative quantification (RQ) values were calculated using the delta-delta method with RQ = $2^{-\Delta\Delta Cq}$ (n=1). The infection types of 0 – 2 were interpreted as resistance (R), and the infection type of 2+ and above were interpreted as susceptible (S).

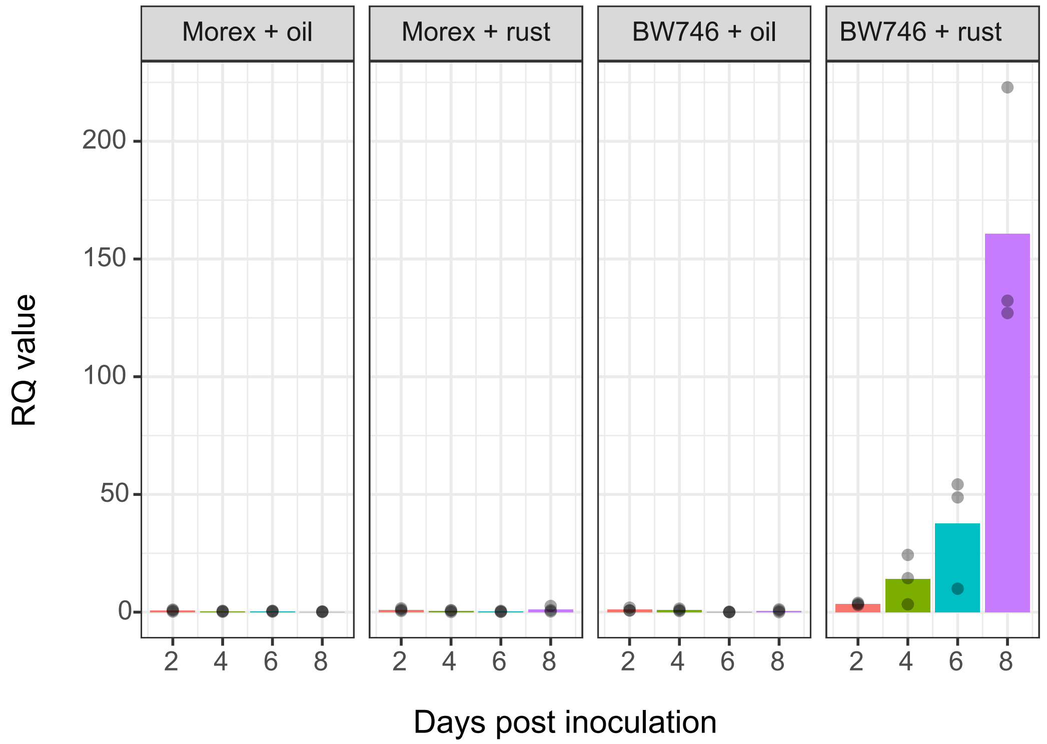

Supplementary Figure 10. Expression profiles for susceptible (cv. Morex, *rph3*) and resistant (BW746, *Rph3*) genotypes when treated with oil alone or challenged with *P. hordei* pathotype 5453 P+. To examine the expression profile of homologs of the *Rph3* gene in susceptible cv. Morex, the marker was designed based on a conserved sequence among four homologs, namely *Morex_ORF5*, *Morex_ORF10*, *Morex_ORF11,* and *Morex_ORF12,* so that it can detect the transcript level of all these genes in total. The expression profile of *Rph3* was examined by using the marker Rph3_qPCR7. The relative quantification (RQ) value was calculated using the delta-delta method with RQ = $2^{-\Delta\Delta Cq}$ (n=3).

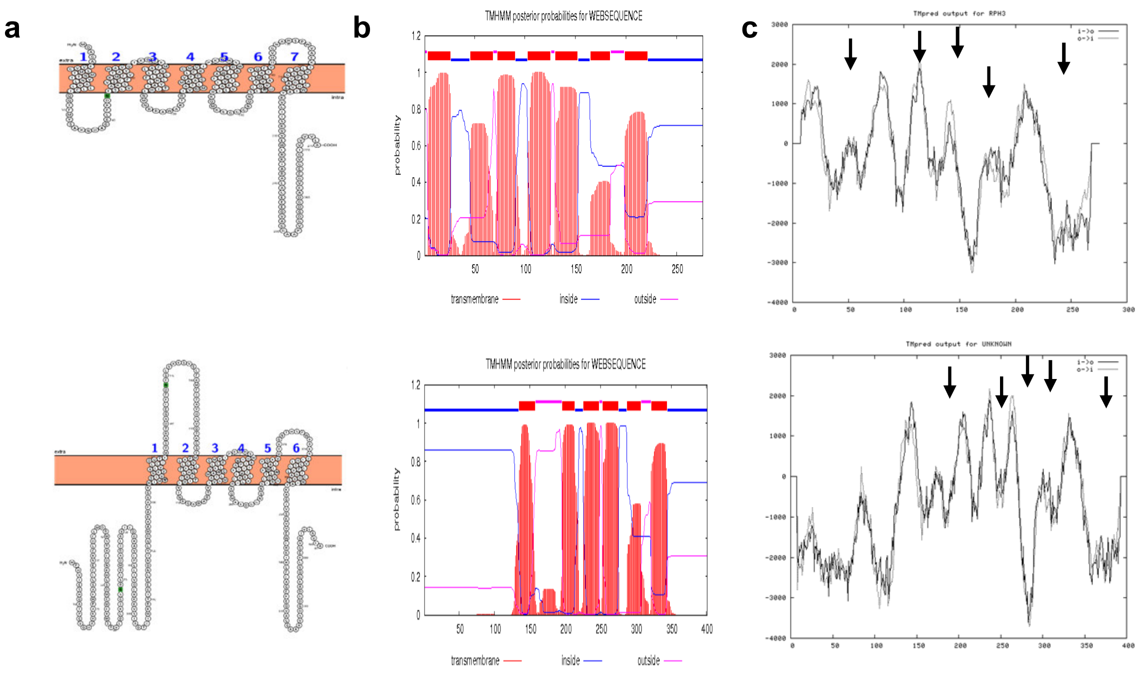

(http://wlab.ethz.ch/protter/start) (http://www.cbs.dtu.dk/services/TMHMM) (https://embnet.vital-it.ch/software/TMPRED_form.html)

Supplementary Figure 11. Predicted secondary structure of RPH3 protein (upper) and its ortholog in the wheat D genome (lower). **a** The prediction made by Protter in which RPH3 protein has seven transmembrane helices, whereas TraesCS7D_RPH3_LIKE has six transmembrane helices. N-glycol motifs are marked in green. Prediction made by the TMHMM (**b**) and TMPRED (**c**) tools; arrows indicate the transmembrane helices.

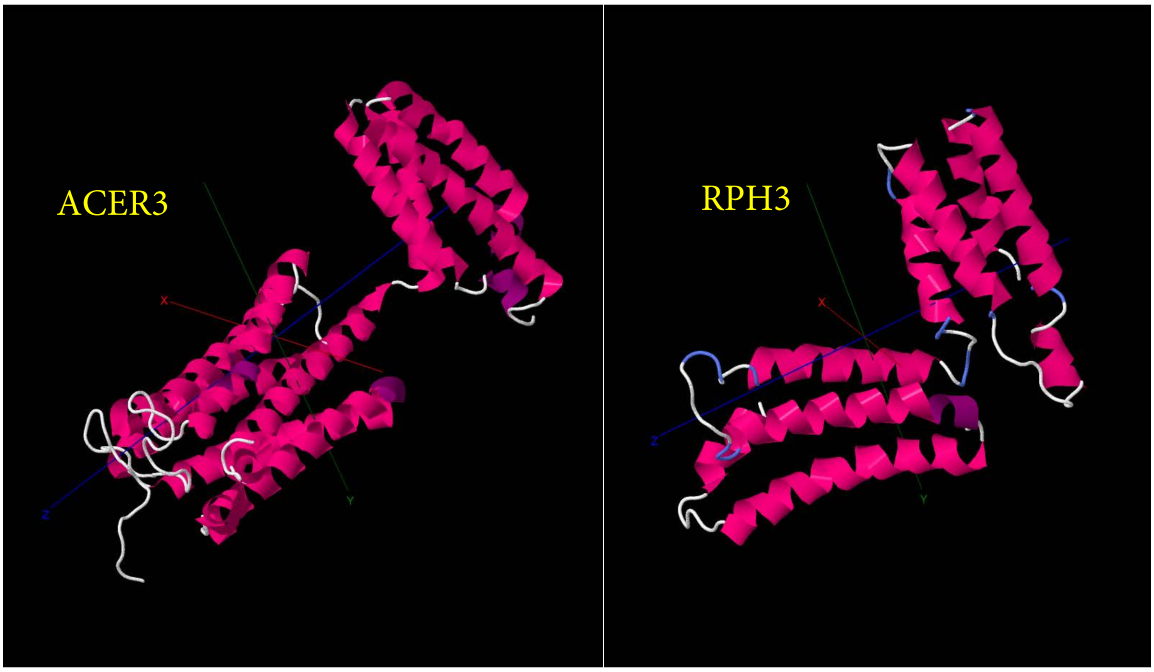

Supplementary Figure 12. Structure of RPH3 compared to homology modelled ACER3 using MODELLER with 6YXH_A as template

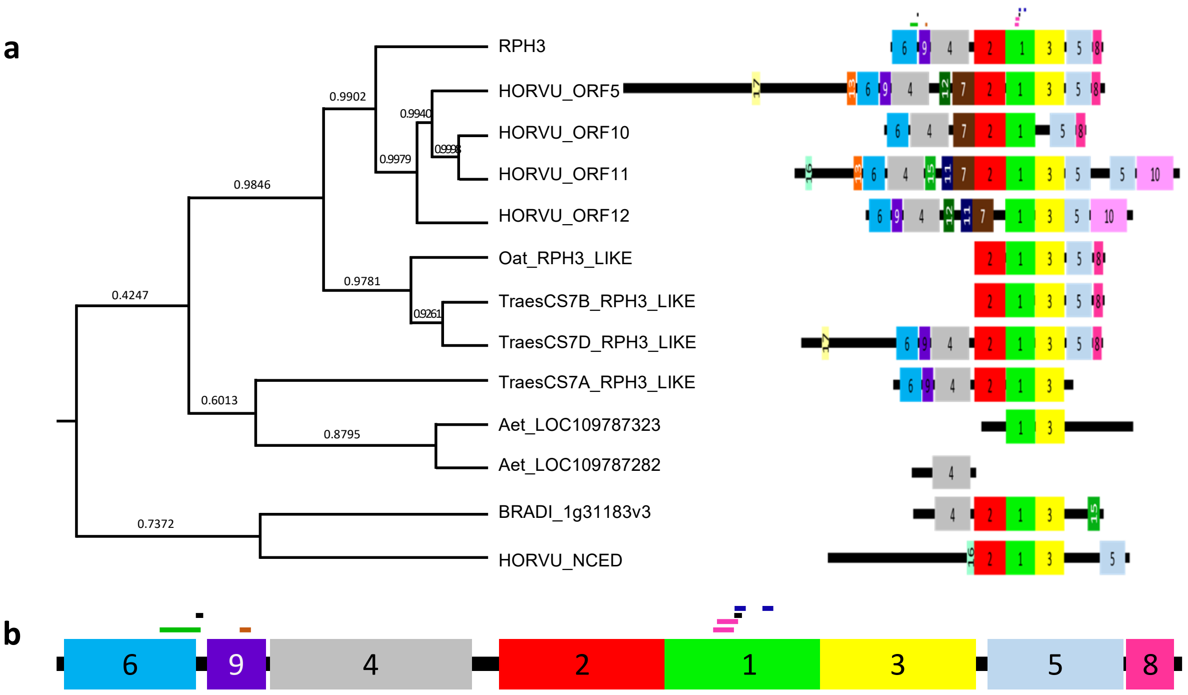

Supplementary Figure 13. Origin of the *Rph3* allele. **a** Comparison of the *Rph3* sequence with *rph3* in cv. Morex and homologs in other species. Four copies of the recessive allele were found in the susceptible haplotype (cv. Morex) encoding four HORVU proteins shown in the phylogenetic tree. The homologs were also found in other crop species, including wheat A, B, and D genomes (three TraesCS proteins), *Brachypodium distachyon* (BRADI protein), and *Aegilops tauschii* (two Aet proteins). The tree was constructed using the maximum likelihood approach based on the protein sequences. The sequences were aligned using Clustal Omega before the phylogenetic analysis. **b** Motifs present in RPH3 homologs. Five homologs of RPH3 in barley include HORVU_NCED sharing 46% identity, HORVU_ORF5 with 72% identity, HORVU_ORF10 with 69% identity, HORVU_ORF11 with 90% identity, and HORVU_ORF12 with 72% identity. Three orthologs of RPH3 on wheat A, B, and D genome (named TRaesCS7A_RPH3_LIKE, TRaesCS7B_RPH3_LIKE, and TRaesCS7D_RPH3_LIKE) share 57%, 88% and 88%identity with RPH3 respectively. Two orthologs from *Aegilops tauschii* (named Aet_LOC109787323 and Aet_LOC109787282) shared 41% and 42% identity with RPH3, respectively. The ortholog in *Brachypodium distachyon* (BRADI_1g31183v3) has 57% identity with RPH3 and Oat_RPH3_LIKE in oat shares 86% identity with RPH3. Motif 1 contains two N-myristoylation sites (pink bars), phosphorylation site of protein kinase C (black bar), and phosphorylation sites of casein kinase II (blue bars), motif 6 contains the serpin signature (green bar), and motif 9 contains N-glucosylation site (brown bar).

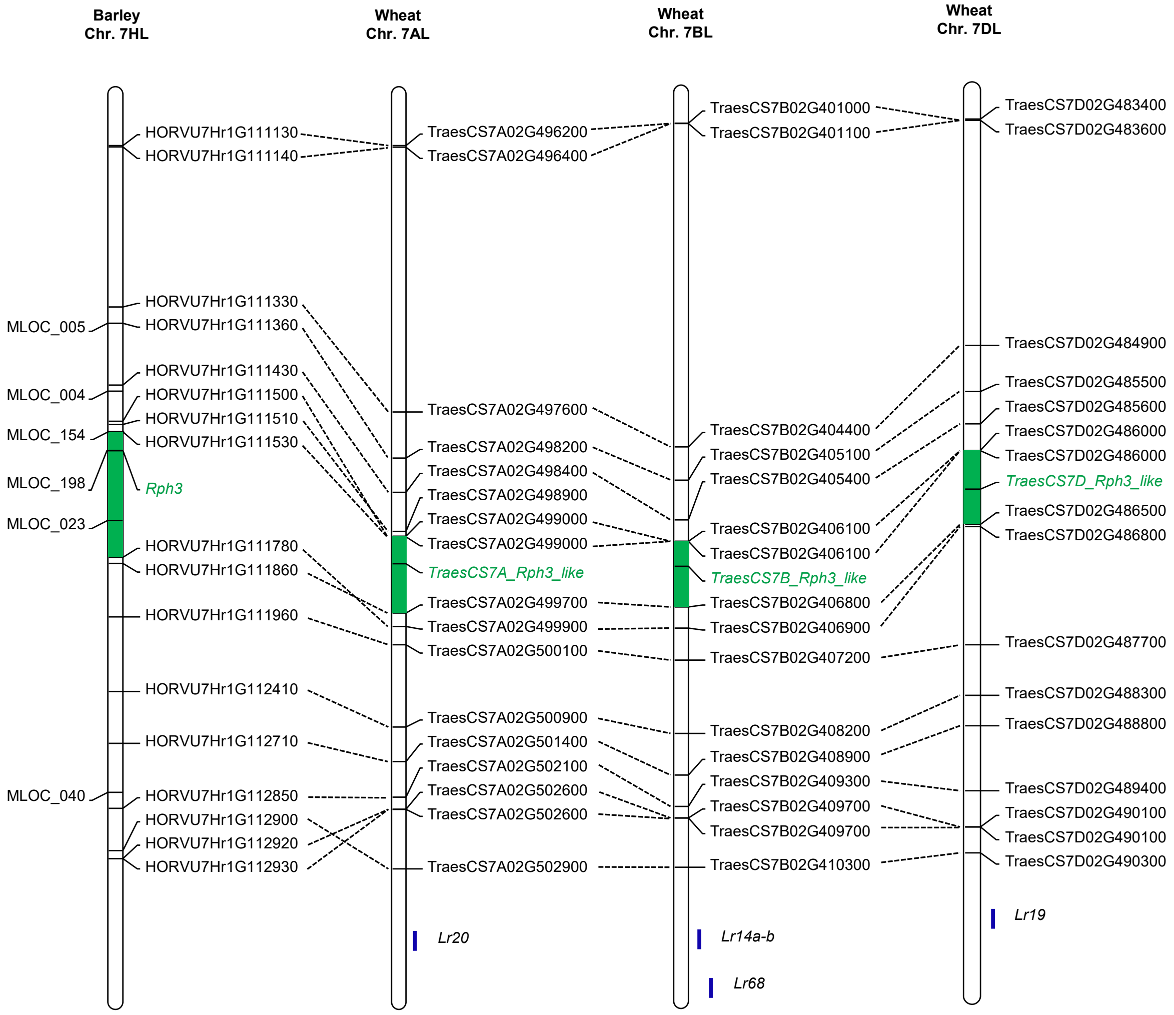

Supplementary Figure 14. Synteny on chromosome 7HL was highly conserved in wheat. Most of the annotated high-confidence genes in barley have their homolog/orthologs in the wheat A, B, and D sub-genomes. The physical windows of the *Rph3* locus are shown as green boxes, and possible orthologs of *Rph3* are green scripts. Four designated wheat leaf rust resistance loci on the long arm of chromosomes 7A, 7B, and 7D are shown by blue bars.

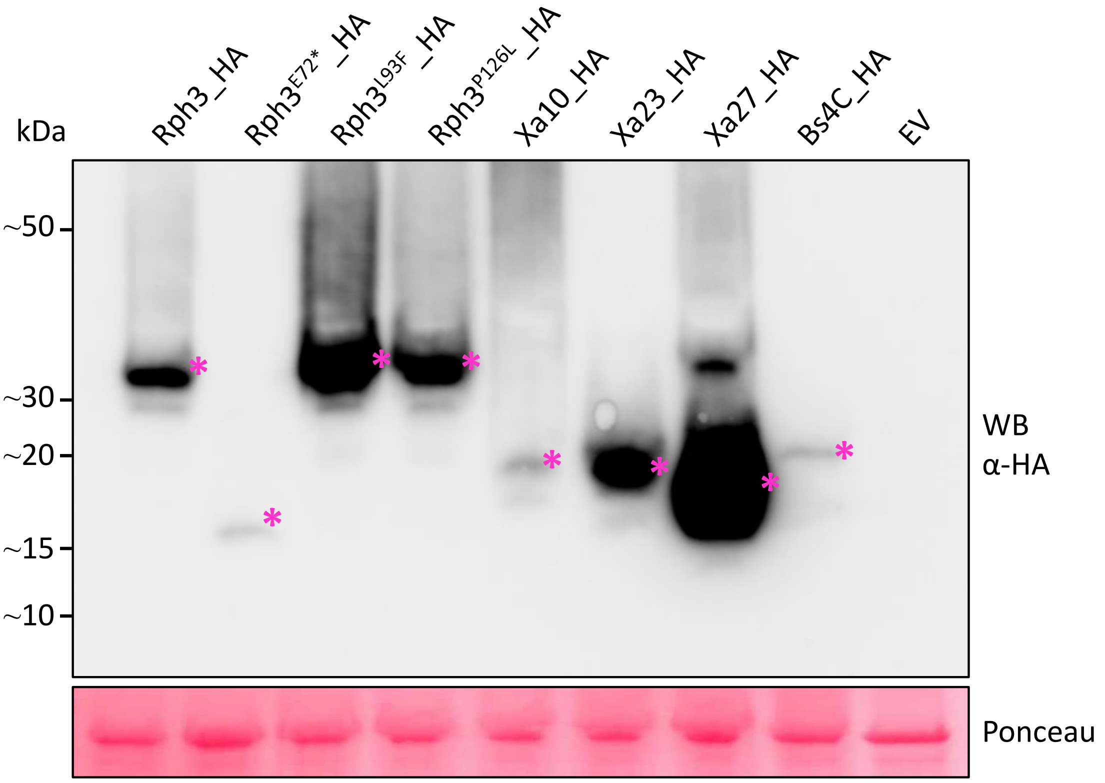

Supplementary Figure 15. Protein accumulation of Rph3 variants and known executor genes. Protein accumulation of RPH3 (37 kDa), RPH3^{E72*} (15 kDa), RPH3^{L93F} (37 kDa), RPH3^{P126L} (37 kDa), and executor genes XA10 (21 kDa), XA23 (20.5 kDa), XA27 (19.5 kDa) and BS4C (27 kDa) in *N. benthamiana* leaves two days after agroinfiltration. All proteins were tagged at the C-terminus with a 6xHA epitope. Asterisks indicate the expected molecular mass for a given recombinant protein. EV corresponds to empty vector control. Protein loading was checked with Ponceau S solution (Sigma, #6226-79-5). Three independent biological replicates were performed with similar results.

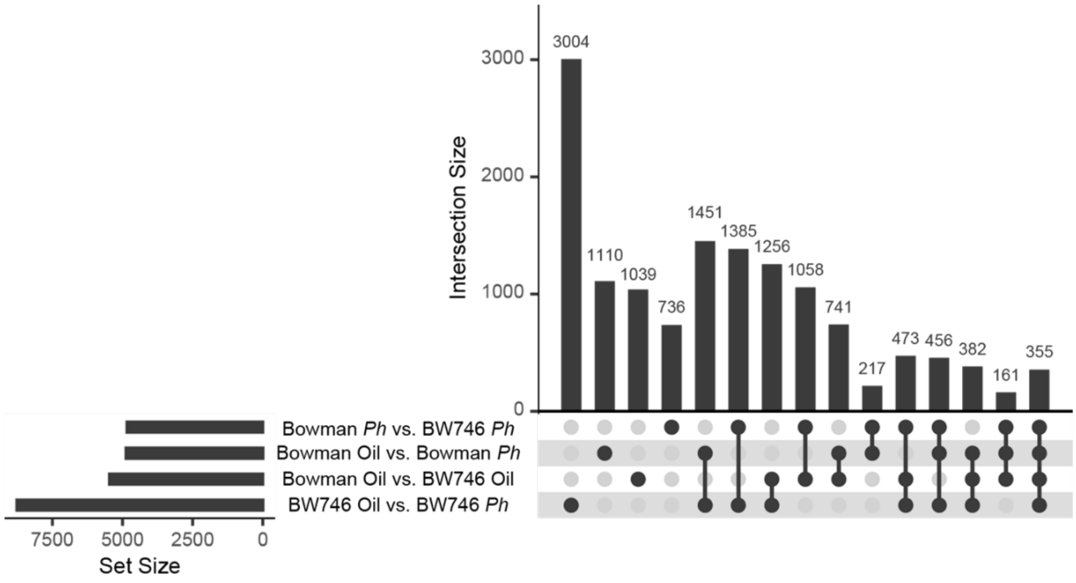

Supplementary Figure 16. Pair-wise comparison of differentially expressed genes identified in cv. Bowman (rph3) and near-isogenic BW746 (Rph3) inoculated with P. hordei or oil alone (mock) at two days post-inoculation. In mock-inoculated conditions, 5,465 differentially expressed genes (DEG) were identified between cv. Bowman and BW746, indicating that a considerable number of genes are differentially expressed between these barley accessions at steady-state levels. Volcano plots showed that most expression differences were minor and likely associated with genetic differences between cv. Bowman and BW746 and their interaction with the oil medium used for mock inoculation. In P. hordei-inoculated leaves, there were 4,841 DEG for cv. Bowman versus BW746, and for mock-inoculated versus P. hordei-inoculated cv. Bowman, there were 4,873 DEG. The number of DEG between mock and P. hordei-inoculated in BW746 was 8,762.

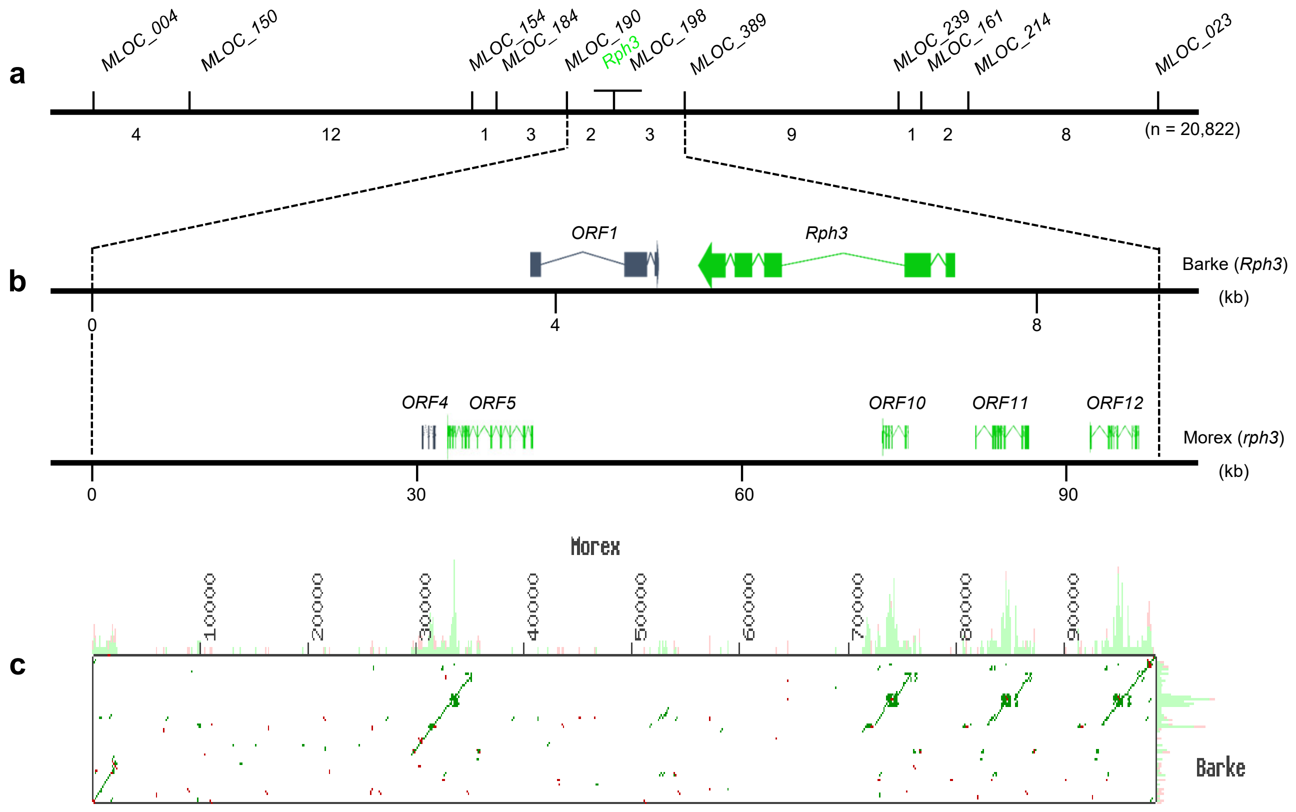

Supplementary Figure 17. Genomic structures of the *Rph3* locus in resistant (Barke) and susceptible (Morex) cultivars. **a** Genetic map of the *Rph3* locus. **b** Physical maps of the *Rph3* locus in cvs. Barke (*Rph3*) and Morex (*rph3*). In cv. Barke, the *Rph3* locus was located in an 8,519-bp region containing putative genes named *ORF1* (black arrow) and *Rph3* (green arrow). In cv. Morex, the same flanked interval was 98,478 bp and contained 12 putative genes named *ORF1* to *ORF12,* among which *ORF4* (black arrow) was a homolog of *ORF1* in cv. Barke and four genes *ORF5*, *ORF10*, *ORF11*, and *ORF12* (green arrows) were homologs of *Rph3*. **c** Dot plot created using the DNA sequence of the *Rph3* locus in cvs. Barke and Morex. The second half of the 8.5-kb DNA fragment in cv. Barke was repeated four times in cv. Morex, where four homologs of *Rph3* were detected.

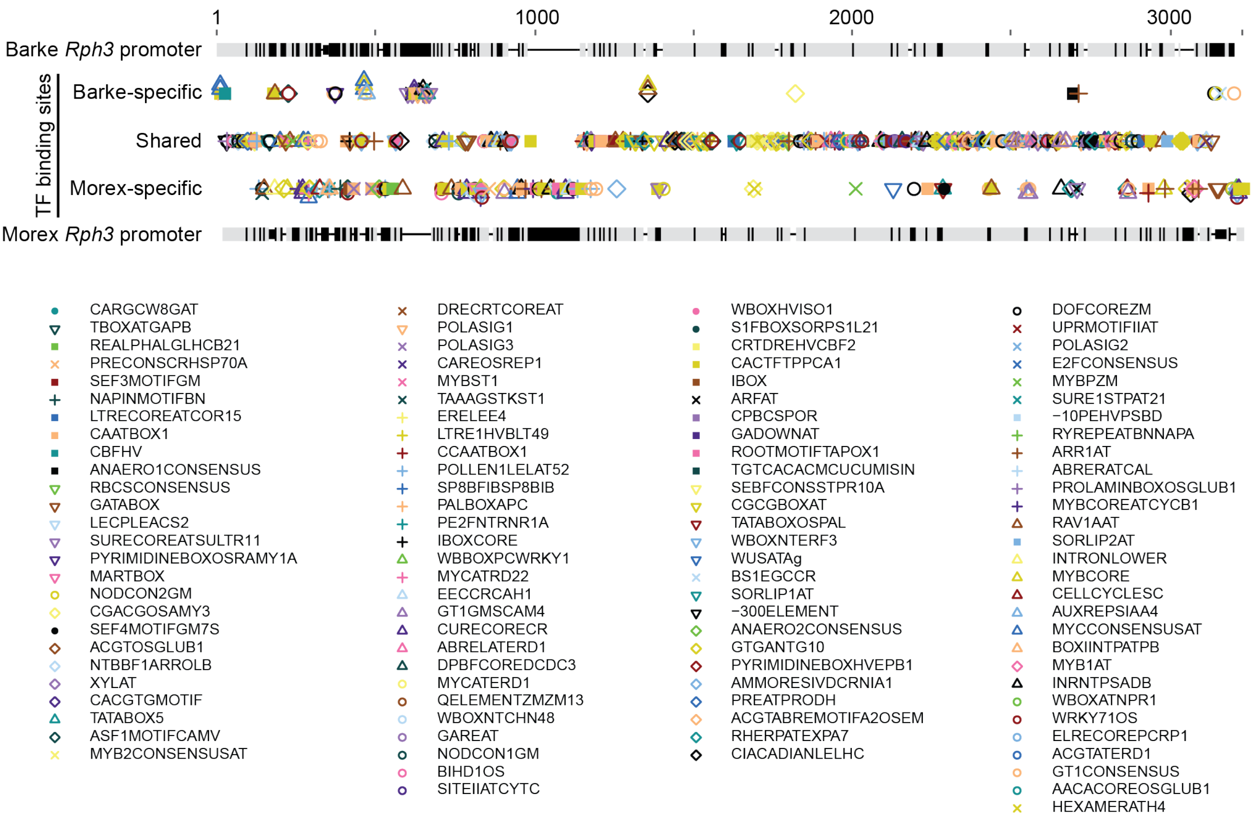

Supplementary Figure 18. Extensive sequence variation in the *Rph3* promoter between Barke (*Rph3*) and Morex (*rph3*) haplotypes. Pairwise alignments of *Rph3* promoters in Barke (*Rph3*) and Morex (*rph3*) were performed using Geneious alignment (gap open penalty of 12; gap extension penalty of 3). Sequence identity was 81.7% (2,598/3,179). Transcription factor binding sites from PLACE (version 30.0) were superimposed on the alignment. Shared, Barke-specific, and Morex-specific binding sites were identified within the conserved and sequence unique regions, respectively. Orientation of the promoter is 5' to 3'. The legend shows the unique color and shape coding for all transcription factor binding sites.

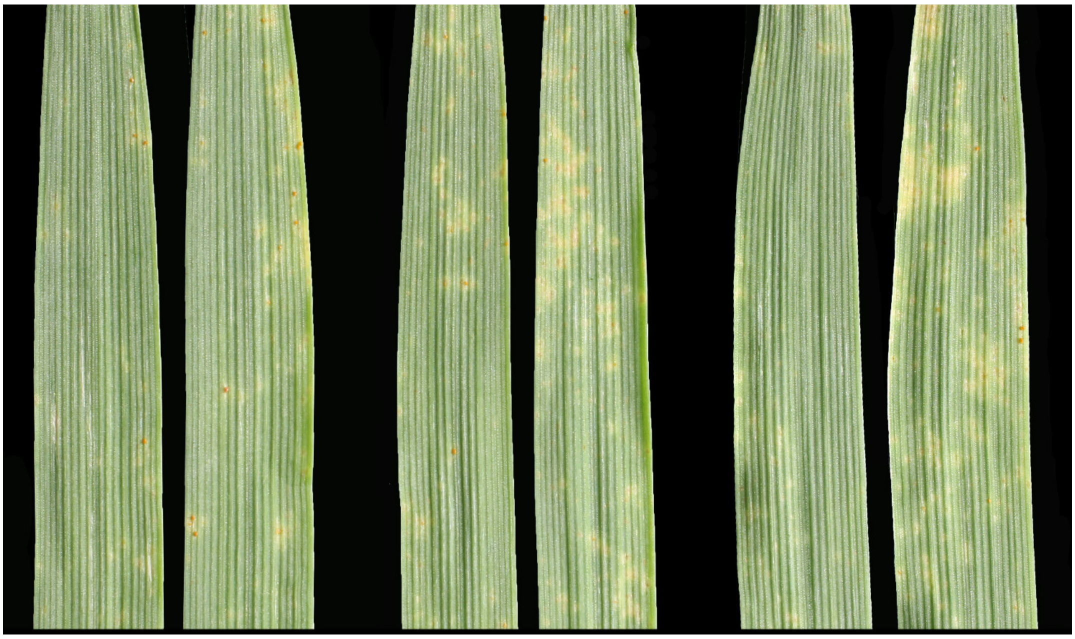

Rph3.c *Rph3.aa* *Rph3.w*

Supplementary Figure 19. The responses of various postulated alleles of the *Rph3* gene to the *P. hordei* pathotype 5453 P+. In all three lines, 86ZBY99 carrying *Rph3.c*, 87ZBY99 carrying *Rph3.aa*, and 88ZBY99 carrying *Rph3.w*, the infection type was similar, with tiny colonies and occasional uredinia surrounded by chlorosis. All resistant parents in mapping populations (Supplementary Table 3) carry the Rph3.c allele. The inoculation was performed on 15 – 20 plants of each material with similar results.

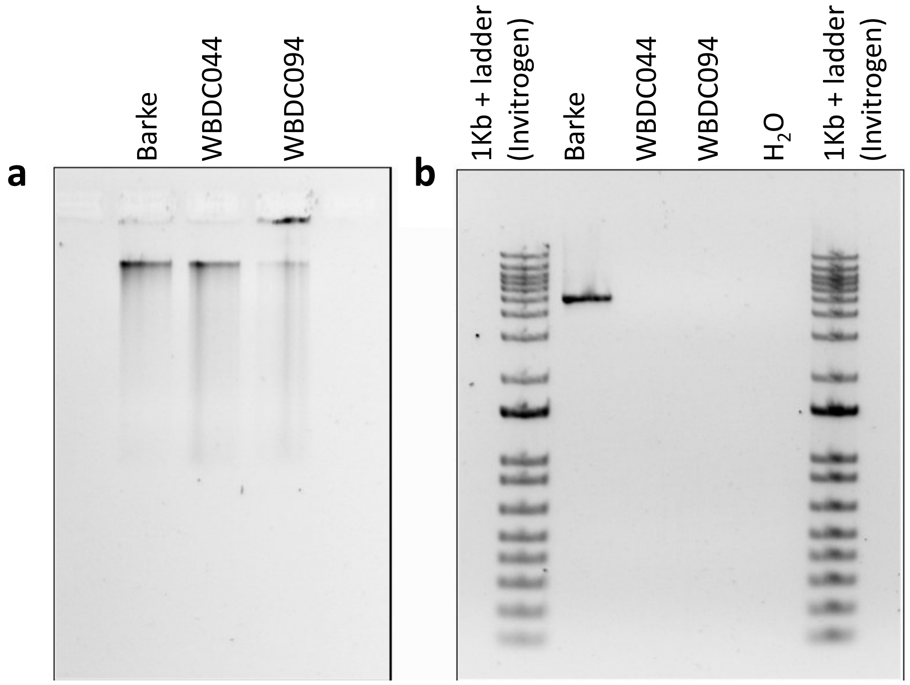

Supplementary Figure 20. The amplification of the *Rph3* alleles in wild barley accessions. **a** Quality of the DNA samples extracted from cv. Barke and two wild barley accessions (WBDC044 and WBDC094). **b** The amplification of the *Rph3* alleles in two wild barley accessions (WBDC044 and WBDC094) with Barke was used as positive control and water was used as no template control.

Supplementary Table 1. The predominance of *P. hordei* pathotype virulent for the *Rph3* gene in Australia

Year	Number of isolates	Number isolates virulent for *Rph3*	Frequency (%) of virulence for *Rph3*
2009	96	6	6.30
2010	172	42	24.40
2011	65	3	4.60
2012	25	0	0.00
2013	53	20	37.70
2014	48	23	47.90
2015	55	31	56.40
2016	116	112	96.60
2017	198	160	80.80
2018	20	16	80.00
2019	10	9	90.00

Supplementary Table 2. Pedigrees and gene postulation of the barley materials used in the construction of genetic map, mutant population, and the complementation test

Cultivar	Synonym/ identifier	Rph3 Genotype	Other *Rph* genes	Pedigree	Response to *P. hordei* pathotype						Used for
					200 P- [=518]	5453 P+ [=584]	5457 P+ [=612]	220 P+ Rph13 [=577]	253 P- [=490]	4610 P+ [=507]	
Alexis	PI 564487	*Rph3*	Nil	Breun1622/Triumph	;cn	;1+cn	3+	;12n	;1n	;12n	Crossing
Barke	HOR 13170	*Rph3*	Nil	Libelle/Alexis	0;n	;1cn	3+	0;=	0;n	0;	Physical mapping
BW746	BW746	*Rph3*	Nil	Bowman*11//Estate/3.2 uz als	0;n	;1-cn	3+	;c	;cn	;c	Crossing and mutating
Henley	-	*Rph3*	Nil	Cellar/NSL97-554	;cn	;1+cn	3+	0;	;cn	;c	Mutating
Scarlett	3880-I	*Rph3*	Nil	Amazone/Breun-2730-E//KYM	;+cn	;1cn	3+	0;=	;n	0;	Crossing
Volla	PI 321852	*Rph3*	Nil	Breuns Wisa/Heines Haisa 1	0;	;1cn	33+	0;=n	;n	0;-	Crossing
Bowman	PI483237	*rph3*	Nil	ND2685/ND1156//Hector	33+	3+	3+	3+	3+	3+	Crossing
Flagship	WI3408	*rph3*	Nil	Chieftan/Barque//Manley/VB9104	3+	33+c	3+	3+	3+	3+	Crossing
Golden Promise	Line 759/4	*rph3*	Nil	gamma-ray induced semi-dwarf mutant of the cultivar 'Maythorpe'	33+	3+	3+	33+	3+	33+	Gene transforming
Gus	PI 494521	*rph3*	Nil	Selection from Composite Cross XXXII-76	3+	3+	3+	3+	3+	3+	Crossing
Sloop	WI2875-22	*rph3*	*Rph19*	RL1577/84/Schooner	1+2+c	3+	3+	3+	;1n	3+	Crossing
Tallon	PI 573731	*rph3*	*Rph12*	Triumph/Grimmett	;1=n	3+	3+	;+n	;1+n	3+	Crossing

Supplementary Table 3. The mapping populations used for high-resolution mapping of the *Rph3* locus

Female (allele)	Male (allele)	Generation	No. plants	No. gametes	No. recombinant plants [b]
Tallon (*rph3*)	Scarlett (*Rph3*)	F2	4,100	8,200	22
Sloop (*rph3*)	Alexis (*Rph3*)	F2	4,553	9,106	15
Volla (*Rph3*)	Gus (*rph3*)	F2	466	932	5
Volla (*Rph3*)	Flagship (*rph3*)	F2	645	1,290	1
Bowman (*rph3*)	BW746(*Rph3*) [a]	F2	553	1,106	2
BW746(*Rph3*) [a]	Gus (*rph3*)	F2	94	188	0
**Total**			**10,411**	**20,822**	**45**

[a] The near-isogenic line of the cultivar Bowman carrying the *Rph3* .c allele from the landrace Estate

[b] The recombinations were between markers MLOC_004 and MLOC_023

Supplementary Table 4. Description of EMS induced mutants in the *Rph3* locus

Mutant lines	Origin	Mutant position	Nucleotide change	Amino acid change	Site of mutation (Exon/Intron number)	Infection type [c]	Fetility status	Observation on development
M167	BW746 [a]	1907	C > T	Leu93Phe	Exon 2	33-	Partially fertile	Well developed, healthy seeds
M181	BW746 [a]	3057	C > T	Pro126Leu	Exon 3	2+	Partially fertile	Well developed, healthy seeds
M466	BW746 [a]	1593	G > A	frameshift mutant	Intron 1	3+	Partially fertile	Very stunted, abortive seeds
M198	Henley	1844	G > T	Glu > Stop codon	Exon 2	33+	Partially fertile	Well developed, healthy seeds
M177	BW746 [a]	None	None	None	Ni [b]	22+c	Partially fertile	well developed, pale green leaves, abortive seeds
M188	BW746 [a]	None	None	None	NI [b]	22+c	Partially fertile	Well developed, healthy seeds

[a] The near-isogenic line of the cultivar Bowman carrying the *Rph3.c* allele from the landrace Estate

[b] No Information

[c] Inoculated with *P. hordei* pathotype 5453P+. The scoring is "0"-"4" known as infection types (IT), conveying variation from complete immunity (0) to a fully susceptible response (4). The letters c and n are included to indicate greater than normal chlorosis or necrosis, respectively. The symbols − is added to indicate infection types that is lower than normal, and + to indicate infection types higher than normal.

Supplementary Table 5. Barley induced mutants selected for *Rph3* using forward genetics

Parent 1 [a]	Parent 2 [a]	Type of cross	Response to pathotype 5453P+ [c]			Allelic status
			Parent 1	Parent 2	F1	
M167	M181	(I) x (I)	33-	2+	22+c	Allelic
M167	M188	(I) x (II)	33-	22+c	;1+c	Not allelic
M177	M188	(II) x (II)	22+c	22+c	;1+c	Not allelic
BW746 [b]			;1-cn			
Bowman			3+			

[a] All the parents used for crossing were derived from BW746

[b] The near-isogenic line of the cultivar Bowman carrying the *Rph3* .c allele from the landrace Estate

[c] The scoring is "0"-"4" known as infection types (IT), conveying variation from complete immunity (0) to a fully susceptible response (4). The letters c and n are included to indicate greater than normal chlorosis or necrosis, respectively. The symbols − is added to indicate infec2on types that is lower than normal, and + to indicate infection types higher than normal.

Group (I) consists of known mutants and group (II) consists of altered phenotype mutants without changes in the *Rph3* sequence

Supplementary Table 6. Progeny test of *Rph3* transgenic plants in barley

Accessions	No. of seedlings	Phenotype (resistance : susceptible)	Genotype of MLOC_400 (*Rph3* : *rph3*)	inconsitency
Estate	12	12 : 0	12 : 0	0/12
BW746	12	12 : 0	12 : 0	0/12
Bowman	10	0 : 10	0 : 10	0/10
Golden Promise	10	0 : 10	0 : 10	0/10
BG_925_E02	10	7 : 3	7 : 3	0/10
BG_925_E04	11	0 : 11	4 : 7	4/11
BG_925_E06	9	6 : 3	6 : 3	0/9
BG_925_E08	12	12 : 0	12 : 0	0/12
BG_925_E10	12	9 : 3	9 : 3	0/12
BG_925_E12	11	0 : 11	0 : 11	0/11
BG_925_E13	10	1 : 9	1 : 9	0/10
BG_925_E15	12	10 : 2	12 : 0	2/12
BG_925_E17	12	0 : 12	0 : 12	0/12
BG_925_E18	10	8 : 2	8 : 2	0/10
BG_925_E19	12	11 : 1	11 : 1	0/12
BG_925_E20	12	0 : 12	0 : 12	0/12
BG_925_E21	13	11 : 2	11 : 2	0/13
BG_925_E22	10	3 : 7	3 : 7	0/10
BG_925_E23	12	9 : 3	9 : 3	0/12
BG_925_E24	11	11 : 0	11 : 0	0/11
BG_925_E25	12	5 : 7	5 : 7	0/12
BG_925_E26	11	5 : 6	5 : 6	0/11
BG_925_E27	10	1 : 9	1 : 9	0/10
BG_925_E28	9	7 : 2	7 : 2	0/9

Supplementary Table 7. The sequences similar to the RPH3 protein identified by the Hidden Markov Model HHpred

Hit	Probability	E-value	P-value	Score (1)	SS (2)	Aligned Cols (3)	Query HMM	Template	HMM	Organism
7B2L_B	54.8	22	0.00036	30.6	3.6	48	143-194	63-110	-291	*Saccharomyces cerevisiae*
5OO7_A	45.9	2.10E+02	0.0034	23.8	7.8	67	143-235	61-127	-257	*Chaetomium thermophilum*
6YXH_A	36.2	3.50E+02	0.0057	23.5	14.3	178	58-239	127-341	-350	*Homo sapiens*
5L26_C	33.7	5.60E+02	0.0092	25.1	14.2	132	69-242	3-143	-431	*Neisseria wadsworthii*
6V7U_A	32.5	2.60E+02	0.0043	22.4	6.1	48	170-240	6-53	-69	*Pseudomonas virus*
1TZV_A	30.5	2.00E+02	0.0032	21.6	5.0	68	167-236	44-114	-142	*Thermotoga maritima*
4R3Q_B	30.4	1.70E+02	0.0027	23.9	4.8	40	195-240	24-66	-88	*Mus musculus*
6FBX_A	27.7	3.50E+02	0.0057	20.9	9.9	86	151-236	20-125	-152	*Danio rerio*
6VN7_R	27.2	6.50E+02	0.011	23.8	13.9	134	73-224	132-269	-582	*Homo sapiens*
6YS8_C	26.9	3.90E+02	0.0064	24.2	7.0	71	67-137	2-73	-215	*Flavobacterium johnsoniae*
2VPZ_G	26.6	5.30E+02	0.0085	22.5	8.8	85	61-152	73-164	-253	*Thermus thermophilus*
7JRG_O	23.0	7.90E+02	0.013	23.3	13.3	121	9-148	39-159	-393	*Vigna radiata var. radiata*
6S7T_C	20.1	4.70E+02	0.0076	19.6	5.6	45	76-120	24-73	-79	*Homo sapiens*
5A1S_D	20.1	7.10E+02	0.011	23.5	7.5	112	33-146	8-127	-448	*Salmonella enterica*

(1) Raw score

(2) Secondary structure score

(3) Length of the aligned region

Supplementary Table 8. GBS markers landing on the *Rph3* gene was used for detecting the gene in the worldwide barley collection

Marker name	Sequence
gRph3_I1E2	GAAATGGATGCAGGAGCTTTCGTCGTCGGCAGTATGACCATGAACACCATCGTGAACTTGGCCGTTCTCCTGCTCTGCATCCAACAGACGTAAGATAAAGAAATGTT
gRph3_E2I2	CGCGGGCGAACTGTTTCTTCGTAATTTTCAGTCGCTCGAGCTTCAAATCTCGTGTTCTACGACAGGCGGTGCTGCTCTCCGTTGTGTGCTCCCACCGCTAACGACAC

Supplementary Table 9. Virulence profile of *P. hordei* and *P. triticina* pathotypes used in this study

Pathotype	Species	Culture number	Year of collection	Place of collection	Virulent
200 P-	*Puccinia hordei*	518	not known	Queensland, Australia	*Rph8*
5453 P+	*Puccinia hordei*	584	2004	Western Australia, Australia	*Rph1-2, Rph4, Rph6, Rph9-10, Rph12, Rph19*
5457 P+	*Puccinia hordei*	612	2009	Queensland, Australia	*Rph1-4, Rph6, Rph9-10, Rph12, Rph19*
5656 P+	*Puccinia hordei*	623	2012	Victoria, Australia	*Rph1-4, Rph6, Rph8-10, Rph12, Rph19*
26-0	*Puccinia triticina*	111	1974	New South Wales, Australia	*None*
104-1,2,3,(6),(7),11,13	*Puccinia triticina*	547	2000	South Australia, Australia	*None*

Supplementary Table 10. Primers used for the expression analysis

Marker name	Target gene	Forward primer	Reverse primer	Annealing temperature	Extension time (sec)	Product size (bp)
Rph3_qPCR7	*Rph3*	CGTCGGCAGTATGACCATGA	GCGCCGAATACATGAACGTC	60	20	83
ORF1_qPCR1	*ORF1* in Barke	TGCCAGGAACAACACACAGT	GGCTCGTTCCTTTCCTTTCT	60	20	113
rph3_qPCR5	*ORF5, ORF10, ORF11 and ORF12* in Morex	AGGATCACTCCTGGCACACT	TTTTGATTGCCTCCATCCTC	60	20	112
ADPRF	ADP-Ribosylation Factor	CCCTGTGGAGGCACTACTTTCA	TCACGCAGCTCATCCTCATTC	60	20	127
