# Supplementary material for: The barley leaf rust resistance gene Rph3 encodes a predicted membrane protein and is induced upon infection by avirulent pathotypes of Puccinia hordei
Source: Nat Commun. 2022 May 2;13:2386. doi: 10.1038/s41467-022-29840-1 (PMC9061838; doi:10.1038/s41467-022-29840-1)
Supplement: Supplementary file 3 — Description of Additional Supplementary Files [file 41467_2022_29840_MOESM3_ESM.pdf]

File Name: Supplementary Data 1
Description: Marker genotypes of the recombinant inbred lines to construct the basic map of Rph3.

File Name: Supplementary Data 2
Description: The oligo sequences of markers used for constructing the high-resolution map of the gene.

File Name: Supplementary Data 3
Description: Marker genotypes for constructing the high-resolution map of the gene.

File Name: Supplementary Data 4
Description: The list of differentially expressed genes identified in the resistance material challenged with Rph3-avirulent pathotype of Puccinia hordei.

File Name: Supplementary Data 5
Description: PCR primers used for amplification and re-sequencing of 8.5kb, the Rph3 locus in resistant haplotype.

File Name: Supplementary Data 6
Description: The presence/absence of the Rph3 gene in the barley core collection determined by molecular markers and multi-pathotype test.

File Name: Supplementary Data 7
Description: List of barley accession of IPK database carrying Rph3 segment detected by GBS marker landing on the gene.

File Name: Supplementary Data 8
Description: List of Wild Barley Diversity Collection (WBDC) provided by University of Minnesota.
