# Supplementary material for: The barley leaf rust resistance gene Rph3 encodes a predicted membrane protein and is induced upon infection by avirulent pathotypes of Puccinia hordei
Source: Nat Commun. 2022 May 2;13:2386. doi: 10.1038/s41467-022-29840-1 (PMC9061838; doi:10.1038/s41467-022-29840-1)
Supplement: Supplementary file 12 — Reporting Summary [file 41467_2022_29840_MOESM12_ESM.pdf]

Corresponding author(s): Dr. Mohammad Pourkheirandish
Prof. Robert F. Park

Last updated by author(s): Mar 15, 2022

Reporting Summary

Nature Portfolio wishes to improve the reproducibility of the work that we publish. This form provides structure for consistency and transparency in reporting. For further information on Nature Portfolio policies, see our Editorial Policies and the Editorial Policy Checklist.

Statistics

For all statistical analyses, confirm that the following items are present in the figure legend, table legend, main text, or Methods section.

n/a	Confirmed	
☐	☒	The exact sample size (*n*) for each experimental group/condition, given as a discrete number and unit of measurement
☐	☒	A statement on whether measurements were taken from distinct samples or whether the same sample was measured repeatedly
☒	☐	The statistical test(s) used AND whether they are one- or two-sided<br>*Only common tests should be described solely by name; describe more complex techniques in the Methods section.*
☐	☒	A description of all covariates tested
☐	☒	A description of any assumptions or corrections, such as tests of normality and adjustment for multiple comparisons
☐	☒	A full description of the statistical parameters including central tendency (e.g. means) or other basic estimates (e.g. regression coefficient) AND variation (e.g. standard deviation) or associated estimates of uncertainty (e.g. confidence intervals)
☒	☐	For null hypothesis testing, the test statistic (e.g. *F*, *t*, *r*) with confidence intervals, effect sizes, degrees of freedom and *P* value noted<br>*Give P values as exact values whenever suitable.*
☒	☐	For Bayesian analysis, information on the choice of priors and Markov chain Monte Carlo settings
☒	☐	For hierarchical and complex designs, identification of the appropriate level for tests and full reporting of outcomes
☐	☒	Estimates of effect sizes (e.g. Cohen's *d*, Pearson's *r*), indicating how they were calculated

Our web collection on statistics for biologists contains articles on many of the points above.

Software and code

Policy information about availability of computer code

Data collection

- Bio-Rad CFX Manager 3.1 was used to collect data from RT-qPCR experiments
- Illumina PE150 was used for RNA sequencing

Data analysis

- BEAST v1.10.4 was used to construct the phylogenetic tree by the maximum likelihood
- Clustal omega in EMBL-EBI (https://www.ebi.ac.uk/Tools/msa/clustalo/) was used to align sequences
- BEAUti v1.10.4 was used to set the parameters
- TreeAnnotator v1.10.4 was used to create the consensus tree
- FigTree v1.4.4 was used to visualize the created tree
- Trimmomatic (v0.39) was used for trimming reads using parameters 'ILLUMINACLIP:TruSeq3-PE.fa:2:30:10 LEADING:5 TRAILING:5 SLIDINGWINDOW:4:15 MINLEN:36'
- Kallisto (v0.46.0) was used to make pseudo alignments using the barley transcriptome (high and low confidence gene models) based on the 2017 genome annotation
- DESeq2 (1.20.0) was used for differential gene expression analysis with default parameters
- MODELLER tool (https://toolkit.tuebingen.mpg.de/tools/modeller) was used for homology modelling

For manuscripts utilizing custom algorithms or software that are central to the research but not yet described in published literature, software must be made available to editors and reviewers. We strongly encourage code deposition in a community repository (e.g. GitHub). See the Nature Portfolio guidelines for submitting code & software for further information.

Data

Policy information about availability of data

All manuscripts must include a data availability statement. This statement should provide the following information, where applicable:

- Accession codes, unique identifiers, or web links for publicly available datasets
- A description of any restrictions on data availability
- For clinical datasets or third party data, please ensure that the statement adheres to our policy

RNAseq data have been deposited in Sequence Read Archives at National Center for Biotechnology Information (NCBI) under BioProject accession number PRJNA731362. The full-length cDNA and genomic sequence of the isolated gene (Rph3) have been deposited in NCBI with the accession number MZ561688 and MZ561689, respectively. Other datasets generated during the current study are available in supplemental data.

Field-specific reporting

Please select the one below that is the best fit for your research. If you are not sure, read the appropriate sections before making your selection.

☒ Life sciences ☐ Behavioural & social sciences ☐ Ecological, evolutionary & environmental sciences

For a reference copy of the document with all sections, see nature.com/documents/nr-reporting-summary-flat.pdf

Life sciences study design

All studies must disclose on these points even when the disclosure is negative.

Sample size	Sample sizes are disclosed within the manuscript. For example; High-resolution mapping: 10,411 F2 individuals.
Data exclusions	No data were excluded from the analyses.
Replication	At least three biological replications were used in every experiment except for the experiments on transgenic materials because each transgenic plant was unique
Randomization	Randomization is considered in all experiments, including experiments in glasshouses and fields.
Blinding	This is not a clinical research

Reporting for specific materials, systems and methods

We require information from authors about some types of materials, experimental systems and methods used in many studies. Here, indicate whether each material, system or method listed is relevant to your study. If you are not sure if a list item applies to your research, read the appropriate section before selecting a response.

Materials & experimental systems

n/a	Involved in the study	
☐	☒	Antibodies
☒	☐	Eukaryotic cell lines
☒	☐	Palaeontology and archaeology
☒	☐	Animals and other organisms
☒	☐	Human research participants
☒	☐	Clinical data
☒	☐	Dual use research of concern

Methods

n/a	Involved in the study	
☒	☐	ChIP-seq
☒	☐	Flow cytometry
☒	☐	MRI-based neuroimaging

Antibodies

Antibodies used	Commercial antibody used: Anti-HA-Peroxidase, High affinity from rat IgG1 (Roche, 12013819001, 1:5000 dilution, LOT 54193500).
Validation	Validation statements of the commercial antibody Anti-HA-Peroxidase is available from the manufacturer at https://www.sigmaaldrich.com/deepweb/assets/sigmaaldrich/product/documents/760/007/12013819001bul.pdf
